# Urinary metal levels and their association with Parkinson’s disease risk: insights from NHANES 2013–2020

**DOI:** 10.3389/fpubh.2025.1439325

**Published:** 2025-03-26

**Authors:** Jia-jie Lv, Xin-yu Li, Cheng-hao Yang

**Affiliations:** ^1^Department of Vascular Surgery, Shanghai Putuo People's Hospital, School of Medicine, Tongji University, Shanghai, China; ^2^Department of Vascular Surgery, Shanghai Ninth People’s Hospital, Shanghai Jiao Tong University, Shanghai, China; ^3^Department of Plastic and Reconstructive Surgery, Shanghai Ninth People's Hospital, Shanghai Jiao Tong University School of Medicine, Shanghai, China

**Keywords:** heavy metals, Parkinson’s disease, the National Health and Nutrition Examination Survey, WQS regression, Bayesian kernel machine regression

## Abstract

**Background:**

Parkinson’s disease (PD) poses a significant public health challenge worldwide, with both genetic predispositions and behavioral factors contributing to its onset and progression. While the precise mechanisms underlying PD remain uncertain, environmental influences are increasingly acknowledged as critical risk factors. This research focused on investigating the relationship between urinary metal levels and the likelihood of developing PD.

**Methods:**

Using data from the National Health and Nutrition Examination Survey (NHANES), urinary levels of nine metals—barium (Ba), cadmium (Cd), cobalt (Co), cesium (Cs), molybdenum (Mo), lead (Pb), antimony (Sb), thallium (Tl), and uranium (Tu)—were measured in a cohort of 3,148 US adults. To examine their association with Parkinson’s disease (PD) risk, multivariate logistic regression, weighted quantile sum (WQS) regression, and quantile regression were employed to evaluate both single and combined metal exposures. Additionally, Bayesian kernel machine regression (BKMR) was utilized to explore the joint effects of these metals, allowing for the assessment of potential nonlinear and non-additive interactions (using the “BKMR” package). Smooth curve fitting was further applied to visualize the nonlinear relationships between urinary metal concentrations and PD risk.

**Results:**

In the single-exposure model, Mo, Tu and Cd were positively correlated with the risk of PD, with odds ratios (OR) ranging from 4.61 to 5.46 (all *p* < 0.05). Mixed-exposure analyses showed a consistent association (OR 1.48, 95% CI 1.06 to 2.06). The metals with the highest weight in the WQS model were Mo (56.79%), Co (34.20%), Ba (3.33%), and Tu (3.27%). In addition, BKMR model analysis showed that most single and mixed metals were positively associated with PD risk. Taken together, the results suggest that metal concentrations can increase the prevalence of PD.

**Conclusion:**

In conclusion, this cross-sectional analysis of NHANES data indicates that higher urinary concentrations of metals including Mo, Cd, and Tu are associated with increased odds of PD among US adults. Mixed exposures to several metals may jointly elevate PD risk in a dose-dependent manner.

## Introduction

Parkinson’s disease (PD) is the most common severe movement disorder worldwide, affecting about 1% of adults over the age of 60 years (1). This represents the main clinical feature of the disease, whose prevalence steadily increases with age (2). Impaired voluntary motor control leads to signs and symptoms such as akinesia, bradykinesia, hypokinesia, postural instability, rigidity, stooped posture, and resting tremor, often accompanied by impaired gait, stiffness of the arms, legs, and trunk, poor balance and coordination, with severe and worsening bilateral vocal cord paralysis (3). Genetic studies have revealed the heterogeneity of PD and provided insights into its pathogenesis and etiology. Occupational and environmental risk factors, as well as dietary habits, are increasingly recognized as important contributors to the development of PD (1, 2). Studies have identified significant associations between PD and exposure to occupational hazards, such as pesticides, herbicides, and heavy metals (e.g., lead and manganese), which are prevalent in agricultural and industrial environments (1). Furthermore, environmental pollutants, including air pollution and certain solvents, have been linked to an elevated risk of PD, likely due to their neurotoxic effects (1). In addition to these external factors, dietary patterns also appear to play a critical role in modulating PD risk (3). For instance, adherence to a Mediterranean diet—characterized by a high intake of antioxidants (e.g., polyphenols from fruits and vegetables) and omega-3 fatty acids—has been associated with neuroprotective effects (3). Conversely, epidemiological evidence suggests that high dairy consumption and reduced uric acid levels may increase the risk of PD (1, 3). These findings underscore the importance of occupational, environmental, and dietary factors in shaping PD susceptibility, highlighting the need for further investigation into these modifiable risk factors (2–4). Epidemiological investigations have provided strong evidence that behavioral and environmental factors play a key role in disease pathogenesis and progression (4–6).

Heavy metals are ubiquitous in various environmental media such as air, soil, drinking water and food (7, 8). Widespread exposure to toxic metals can lead to diseases such as diabetes and cancer (9, 10). Environmental metal exposure has emerged as one of the most important public health issues globally (11). Among these factors, heavy metal exposure is one of the concerns regarding the pathogenesis of PD. Possible mechanisms by which metals play a role in PD pathogenesis and progression include mitochondrial dysfunction and oxidative stress, promotion of α-synuclein aggregation and fibril formation, and microglial activation and inflammation (12, 13). Recent studies have suggested that inhaling manganese from mining and welding fumes may induce PD, and dental amalgam filling restorations are associated with an increased risk of PD (14). A meta-analysis showed that PD patients had lower serum copper and iron concentrations, while zinc concentrations in serum/plasma and magnesium concentrations in CSF were higher compared to controls (15). Cumulative lead levels in bone have been associated with an increased risk of PD (16). Previous research has predominantly focused on the relationship between PD prevalence and individual metals. However, humans are typically exposed to multiple heavy metals simultaneously, and the combined effects of metal mixtures can differ significantly from those of single metals. This is due to potential synergistic, antagonistic, or other interactive effects that metals may have on human health.

To address this gap, we conducted a cross-sectional study using data from the 2013–2020 NHANES. The study aimed to examine the association between urinary concentrations of nine metals and the risk of PD.

## Methods

### Study population

**Figure 1:**
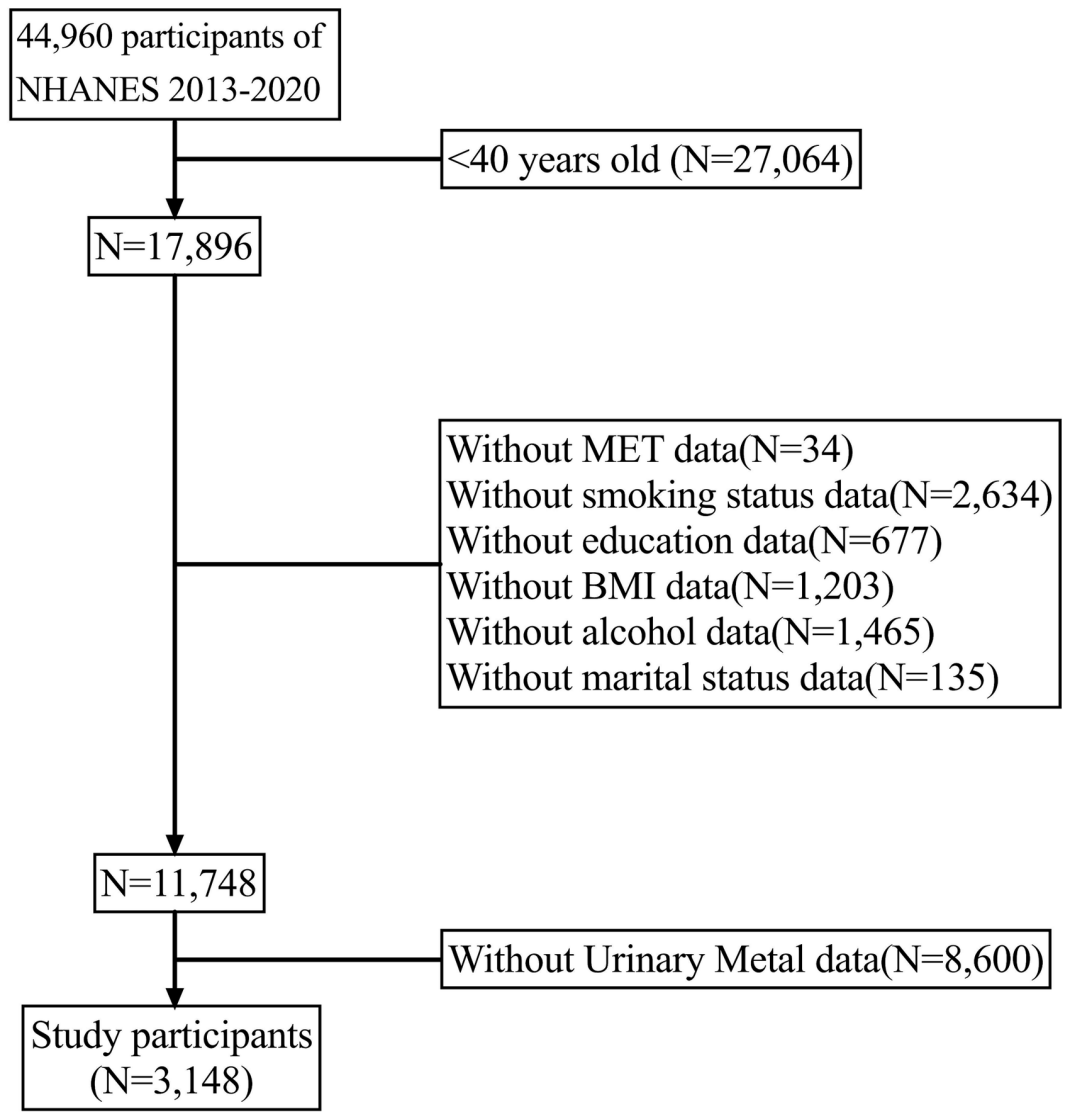
Flow chart of the study.

The National Health and Nutrition Examination Survey (NHANES) is a comprehensive CDC program that evaluates the health and nutrition status of U.S. residents through an interdisciplinary survey. The overarching goal of NHANES is to gather, analyze, and publish data on the health, nutrition, and environmental exposures of the U.S. population. Conducted annually since the 1960s, NHANES surveys individuals of all ages nationwide. To generate more precise estimates and reduce sampling error, we combined four survey periods (2013–2014, 2015–2016, 2017–2018, and 2019–2020) for analysis. In the current study, PD patients were identified per assessment (*n* = 44,960). We then excluded those missing metal exposure data (*n* = 8,600). Ultimately, 3,148 participants were included (Figure 1).

### PD assessment

In this study, participants with PD were identified through the “Second Level Category Name” listed as “ANTIPARKINSON AGENTS” in the Prescription Medications document. This classification was based on participants’ responses to questions about their prescription medication use. Since this method relied on the medications and codes provided in the NHANES dataset, individuals were classified as having PD only if they were actively receiving treatment for the condition. Participants who did not report using anti-parkinsonian medications were categorized as not having PD. The definition of PD used in this study aligns with those used in previous research (16, 17).

### Measurement of urinary metal levels

Detection data for nine urinary metals were sourced from NHANES 1999–2016, including barium (Ba), cadmium (Cd), cobalt (Co), cesium (Cs), molybdenum (Mo), lead (Pb), antimony (Sb), thallium (Tl), and tungsten (Tu). These concentrations were measured primarily from spot urine samples using inductively coupled plasma mass spectrometry (ICP-MS). For values below the limit of detection (LOD), the data were imputed by replacing them with the LOD divided by the square root of two. The quality assurance and control procedures implemented in NHANES adhered to the standards mandated by the 1988 Clinical Laboratory Improvement Act.

### Covariates

Building on prior research and clinical insights, this study considered various sociodemographic and health-related characteristics, including age, sex, race (e.g., Mexican American, White, Black), education level (less than high school, high school graduate, or higher education), marital status (e.g., living with a partner, single, or married), and the poverty income ratio (PIR) (16, 17). PIR, a socioeconomic status indicator, compares total household income to the poverty line and is categorized as low (PIR < 1.35), medium (1.35 ≤ PIR < 3.0), or high (PIR ≥ 3.0). Smoking and alcohol consumption statuses were also evaluated. Smoking status was classified as never smokers (fewer than 100 cigarettes smoked in a lifetime), former smokers (smoked 100 or more cigarettes but no longer smoke), and current smokers (actively smoking and with a lifetime total of at least 100 cigarettes). For alcohol consumption, never drinkers were those who consumed fewer than 12 drinks in their lifetime, while former drinkers consumed more than 12 drinks but had not drunk in the past year. Current drinkers were further categorized into light, moderate, or heavy drinkers. Binge drinking was defined as three or more drinks per day for women, four or more for men, or five or more drinking episodes per month. Moderate drinkers were defined as having two drinks daily for women, three for men, and binge drinking no more than twice a month. Additional health-related factors included body mass index (BMI), metabolic equivalent (MET), and comorbidities such as diabetes, congestive heart failure, coronary artery disease, chronic obstructive pulmonary disease (COPD), hypertension, and cancer. Each comorbidity was scored from 1 to 6 based on its severity and impact on health, and a cumulative comorbidity index (CCI) was calculated by summing the scores, with higher values indicating more severe disease burden. Sociodemographic and health data were gathered using standardized protocols during structured interviews conducted by trained personnel. Demographic details (e.g., age, sex, race, and socioeconomic status) were self-reported by participants, while health-related variables such as BMI (calculated from height and weight measurements) and smoking and alcohol usage were obtained through direct interviews and physical assessments. Environmental exposure data, including levels of metals such as cadmium, cesium, and lead, were derived from laboratory analyses of biological samples (e.g., blood and urine) collected by trained medical staff following established guidelines.

### Statistical analysis

This study utilized R version 4.3.0 for statistical analyses, employing chi-square tests and t-tests to compare demographic characteristics by PD status. Metal concentrations were natural log-transformed (Ln-transformed) for normalization and categorized into quartiles (Q1–Q4). Multivariate logistic regression was used to estimate odds ratios (ORs) and 95% confidence intervals (CIs) for the association between urinary metals and PD risk, adjusting for covariates such as age, sex, race/ethnicity, education, marital status, BMI, MET, alcohol consumption, and poverty-income ratio (PIR). Multicollinearity was assessed using Variance Inflation Factor (VIF), and Pearson correlation analysis was conducted to examine relationships between Ln-transformed metals. Weighted Quantile Sum (WQS) regression, using the “gWQS” R package, was applied to evaluate the combined effects of metal mixtures, with the WQS index representing the simultaneous influence of mixed metal exposure on PD risk.

This study first utilized weighted multiple logistic regression to evaluate the association between urinary metals and PD, constructing three models: the crude model (unadjusted), Model 1 (adjusted for age, sex, and race), and Model 2 (further adjusted for demographic factors such as BMI, PIR, MET, and education, as well as CCI and lifestyle factors like smoking and alcohol consumption). To account for potential nonlinear and non-additive relationships between metals, Bayesian Kernel Machine Regression (BKMR) was applied (using the “BKMR” R package) to assess the combined effects of all metals and the dose–response relationships between individual metals and PD, while varying the concentrations of others. This analysis used the quasi-Bayesian Monte Carlo method with 5,000 simulations based on a normal approximation. Lastly, smooth curve fitting (penalty spline method) was performed to explore nonlinear relationships between urinary metals and PD. All statistical analyses were conducted using R version 4.3.0, with a significance threshold set at *p* < 0.05.

## Results

### Characteristics of participants and metals distribution

Among 3,148 adults, 50 were diagnosed with PD. Table 1 presents the demographic characteristics of the study participants with or without PD. Overall, race, PIR, BMI, CCI, and MET were statistically significant between PD and non-PD participants. The distribution of metal concentrations is shown in Supplementary Table S1. The detection rate of metal was 100%. Pearson coefficients between Ln transition metals showed a moderate correlation between Cs and Tl (r = 0.62), while the other correlations were relatively poor (Supplementary Figure S1). What’s more, to evaluate potential multicollinearity among the independent variables, we performed a VIF test. The results are summarized in Supplementary Table S2. All variables exhibited VIF values below the commonly accepted threshold of 10, indicating no severe multicollinearity issues. These results suggest that each predictor contributes independently to the model, ensuring the robustness of the regression analysis.

**Table 1:** Characteristics of participants by Parkinson’s disease status, NHANES 2013–2020.

Variable	Total	Non-Parkinson	Parkinson	*p*
Age, *n* (%)				0.16
40–54	1,130(35.9)	1,116(40.91)	14(29.95)	
55–66	1,034(32.85)	1,022(34.28)	12(48.11)	
67–80	984(31.26)	960(24.82)	24(21.94)	
Gender, *n* (%)				0.69
Female	1,593(50.6)	1,570(53.08)	23(56.90)	
Male	1,555(49.4)	1,528(46.92)	27(43.10)	
Race/ethnicity, *n* (%)				0.05
Mexican American	351(11.15)	349(6.83)	2(1.41)	
Non-Hispanic Black	822(26.11)	811(10.91)	11(8.53)	
Non-Hispanic White	1,128(35.83)	1,095(65.52)	33(83.09)	
Other Hispanic	293(9.31)	290(6.31)	3(6.47)	
Other race/ethnicity	554(17.6)	553(10.43)	1(0.50)	
Marital status, *n* (%)				0.15
Married/cohabiting	1930(61.31)	1906(67.34)	24(51.53)	
Widowed/divorced/separated	928(29.48)	914(25.95)	14(31.58)	
Never married	290(9.21)	278(6.70)	12(16.89)	
Education level, *n* (%)				0.53
Under high school	665(21.12)	656(12.40)	9(8.25)	
High school or equivalent	799(25.38)	789(28.47)	10(37.60)	
Above high school	1,684(53.49)	1,653(59.13)	31(54.15)	
Alcohol intake, *n* (%)				0.21
Heavy	362(11.5)	360(12.62)	2(1.41)	
Mild	2017(64.07)	1981(63.34)	36(70.24)	
Moderate	473(15.03)	468(17.61)	5(18.84)	
Never	296(9.4)	289(6.44)	7(9.51)	
PIR, *n* (%)				0.01
<1.3	724(26.43)	714(16.53)	10(8.31)	
1.3–3.5	1,088(39.72)	1,065(34.79)	23(59.22)	
>3.5	927(33.84)	913(48.68)	14(32.47)	
BMI, *n* (%)				<0.0001
<18.5	29(0.94)	27(0.49)	2(17.93)	
18.5–28	1,312(42.53)	1,301(39.47)	11(30.56)	
>28	1744(56.53)	1712(60.04)	32(51.50)	
Smoking status, *n* (%)				0.95
Former	897(28.49)	880(29.81)	17(32.40)	
Never	1732(55.02)	1707(56.52)	25(53.89)	
Now	519(16.49)	511(13.67)	8(13.71)	
CCI (SEx)	1.13(0.05)	1.12(0.05)	1.80(0.35)	0.05
MET (SEx)	4937.23(188.62)	4973.29(187.64)	2373.54(380.52)	<0.0001

a. Values are standard error (SEx) for continuous variables and weighted percentage for categorical variables; *p* values were derived from Student’s t test for continuous variables and the Chi-square test for categorical variables. b. PIR: Poverty to income ratio; BMI: Body mass index; CCI: co-morbidity index; MET: metabolic equivalent; NPAR: neutrophil-to-albumin ratio; NLR: neutrophil-to-lymphocyte ratio; FLI: fatty liver index.

### Associations between metal concentration and PD risk

Table 2 summarizes the association between LN-transformed urinary metal concentrations and PD risk as analyzed through weighted multiple logistic regression models. The models adjusted for potential confounders, including age, sex, PIR, education, marital status, BMI, alcohol consumption, MET, CCI, and smoking status. Compared to the lowest exposure quantile (Q1), the highest exposure quantiles of Tu (OR 5.46, 95% CI 1.30–22.87), Mo (OR 5.42, 95% CI 0.93–31.45), and Cd (OR 4.61, 95% CI 1.68–12.61) were significantly associated with an increased risk of PD (all *p* for trend <0.05). Additionally, exposure to mixed metals was positively associated with PD risk (OR 5.12, 95% CI 0.95–27.57), further highlighting the role of metal mixtures in PD development.

**Table 2:** Univariate and multivariate analyses by the weighted linear model.

	Crude Model			Model 1			Model 2		
Exposure	OR	95%CI	*p*	OR	95%CI	*p*	OR	95%CI	*p*
Ba									
Continuous	0.88	0.70,1.11	0.27	0.87	0.66,1.13	0.29	0.89	0.49,1.65	0.71
Q1	Ref			Ref			Ref		
Q2	0.54	0.15,1.89	0.32	0.54	0.15,2.01	0.35	0.57	0.07,4.28	0.57
Q3	1.62	0.67,3.95	0.28	1.57	0.57,4.34	0.37	1.11	0.27,4.58	0.89
Q4	0.40	0.15,1.08	0.07	0.38	0.13,1.12	0.08	0.45	0.06,3.48	0.43
*p* for trend	0.45			0.41			0.57		
Cd									
Continuous	1.13	0.81,1.58	0.46	1.17	0.81,1.69	0.40	0.40	0.82,2.25	0.22
Q1	Ref			Ref			Ref		
Q2	0.88	0.22,3.57	0.86	0.88	0.20,3.94	0.86	0.21	0.05,0.95	0.04
Q3	0.16	0.04,0.60	0.01	0.16	0.04,0.60	0.01	0.08	0.01,0.56	0.01
Q4	1.96	1.08,3.58	0.03	2.20	1.13,4.29	0.02	4.61	1.68,12.61	0.005
*p* for trend	0.04			0.03			0.03		
Co									
Continuous	1.04	0.81,1.34	0.75	1.03	0.80,1.33	0.79	0.99	0.58,1.68	0.96
Q1	Ref			Ref			Ref		
Q2	0.72	0.18,2.87	0.63	0.70	0.18,2.83	0.61	0.38	0.04,3.39	0.37
Q3	1.93	0.82,4.56	0.13	1.93	0.79,4.77	0.15	1.19	0.43,3.27	0.73
Q4	0.67	0.18,2.42	0.53	0.67	0.18,2.48	0.54	0.54	0.08,3.79	0.52
*p* for trend	0.93			0.91			0.81		
Cs									
Continuous	0.57	0.38,0.87	0.01	0.58	0.39,0.88	0.01	0.68	0.39,1.19	0.17
Q1	Ref			Ref			Ref		
Q2	0.25	0.12,0.54	<0.001	0.23	0.11,0.49	<0.001	0.11	0.02,0.68	0.02
Q3	0.28	0.12,0.68	0.01	0.29	0.13,0.67	0.005	0.51	0.12,2.25	0.36
Q4	0.30	0.11,0.84	0.02	0.31	0.11,0.82	0.02	0.58	0.28,1.19	0.13
*p* for trend	0.02			0.02			0.35		
Mo									
Continuous	1.92	1.42,2.61	<0.0001	2.05	1.35,3.11	0.001	2.12	1.22,3.68	0.01
Q1	Ref			Ref			Ref		
Q2	1.87	0.45,7.81	0.38	1.89	0.42,8.43	0.39	3.19	0.66,15.45	0.14
Q3	1.87	0.61,5.77	0.27	2.05	0.59,7.19	0.25	1.82	0.28,11.94	0.52
Q4	5.52	1.94,15.70	0.002	6.40	1.73,23.66	0.01	5.42	0.93,31.45	0.05
*p* for trend	0.002			0.005			0.04		
Pb									
Continuous	1.07	0.79,1.43	0.66	1.09	0.78,1.53	0.58	1.33	0.80,2.20	0.26
Q1	Ref			Ref			Ref		
Q2	4.61	1.52,14.00	0.01	5.23	1.37,19.91	0.02	2.48	0.55,11.28	0.23
Q3	2.77	0.98,7.78	0.05	3.00	0.85,10.57	0.08	2.73	0.57,13.10	0.20
Q4	1.63	0.64,4.15	0.29	1.86	0.65,5.30	0.24	1.60	0.40,6.43	0.49
*p* for trend	0.49			0.40			0.30		
Sb									
Continuous	0.68	0.42,1.08	0.10	0.68	0.42,1.12	0.12	0.53	0.22,1.28	0.15
Q1	Ref			Ref			Ref		
Q2	2.13	1.02,4.45	0.04	2.23	0.91,5.46	0.08	1.10	0.42,2.92	0.84
Q3	0.28	0.06,1.20	0.08	0.30	0.07,1.32	0.11	0.30	0.05,1.79	0.18
Q4	0.68	0.20,2.31	0.52	0.72	0.19,2.69	0.62	0.39	0.06,2.39	0.29
*p* for trend	0.10			0.13			0.16		
Tl									
Continuous	0.66	0.37,1.19	0.16	0.69	0.39,1.23	0.20	1.40	0.58,3.34	0.44
Q1	Ref			Ref			Ref		
Q2	0.18	−0.24,0.06	<0.0001	0.17	−0.17,0.07	<0.0001	0.16	−0.18,0.07	0.04
Q3	0.18	−0.21,0.07	<0.0001	0.17	−0.14,0.12	<0.0001	0.30	−0.14,0.12	0.13
Q4	0.24	−0.15,0.08	0.01	0.25	−0.05,0.14	0.01	0.70	−0.05,0.14	0.04
*p* for trend	0.01			0.01			0.04		
Tu									
Continuous	1.45	1.23,1.70	<0.0001	1.51	1.20,1.91	<0.0001	1.94	1.19,3.16	0.01
Q1	Ref			Ref			Ref		
Q2	0.97	0.26,3.63	0.96	1.05	0.29,3.83	0.93	1.88	0.31,11.48	0.48
Q3	7.73	4.14,14.43	<0.0001	7.92	3.91,16.05	<0.0001	12.40	3.17,48.49	<0.001
Q4	2.32	1.02,5.27	0.05	2.54	1.10,5.83	0.03	5.46	1.30,22.87	0.02
*p* for trend	<0.0001			<0.0001			0.003		
Mixed Metals									
Continuous	1.80	1.28,2.52	0.001	1.90	1.22,2.97	0.01	1.99	1.12,3.54	0.02
Q1	Ref			Ref			Ref		
Q2	1.89	0.49,7.30	0.35	2.02	0.49,8.30	0.32	3.29	0.73,14.83	0.11
Q3	1.84	0.59,5.71	0.28	2.02	0.56,7.36	0.27	1.87	0.27,13.21	0.51
Q4	5.19	1.84,14.65	0.003	5.97	1.64,21.70	0.01	5.12	0.95,27.57	0.05
*p* for trend	0.004			0.01			0.04		

a. Crude Model: no covariates were adjusted. b. Model 1: adjusted for age; race. c. Model 2: adjusted for age; race; poverty: income ratio; education level; marital status; BMI; smoking; CCI.

**Figure 2:**
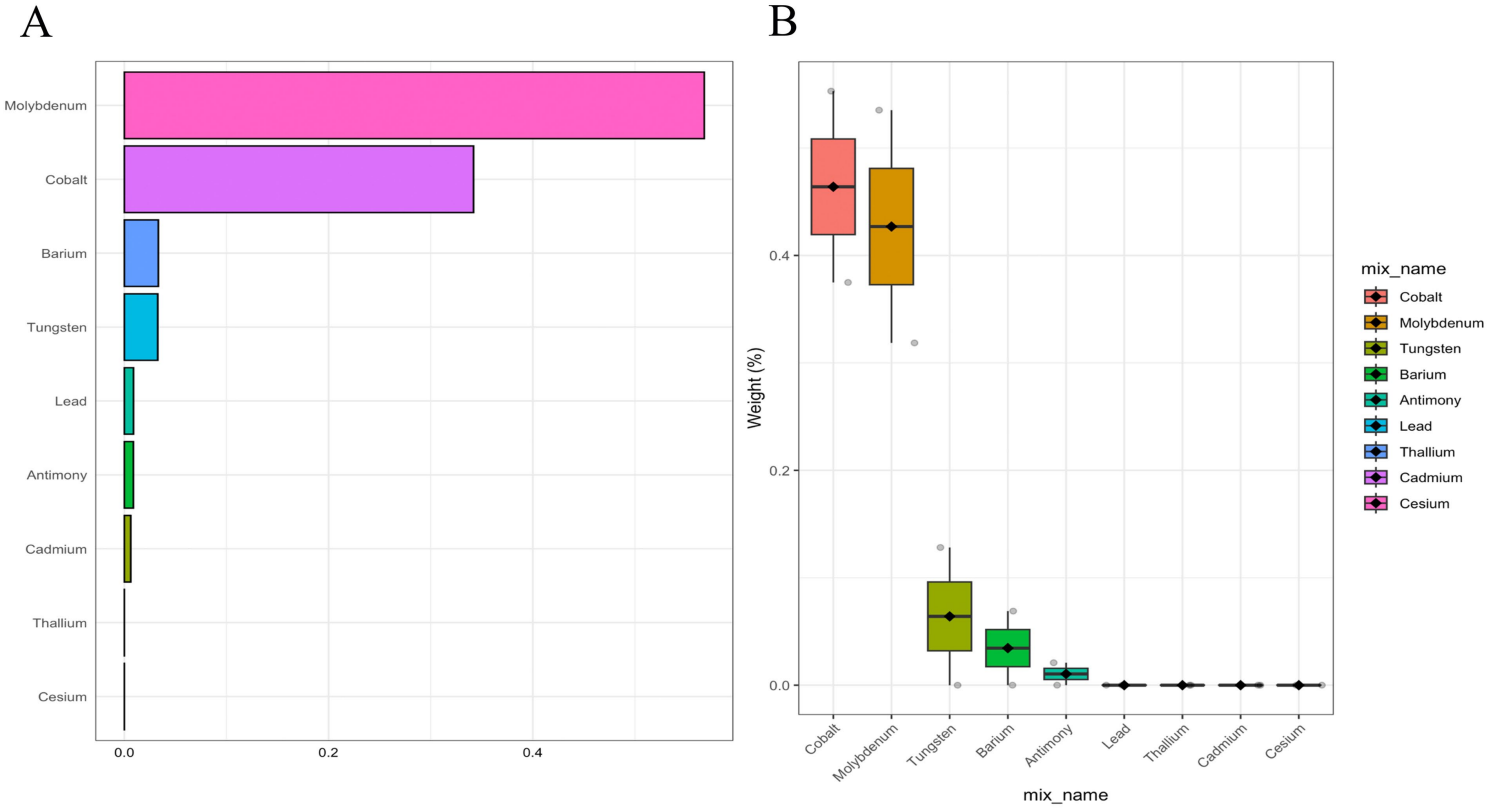
Weighted contributions of urinary metals to Parkinson’s Disease in WQS models adjusted for demographics, lifestyle, and health indicators (gender, age, race, education, PIR, marital status, BMI, MET, drinking alcohol status, smoking status, and CCI). **(A)** Bar plot showing the weighted contributions of urinary metals. **(B)** Box plot showing the distribution of weights for urinary metals by category.

**Figure 3:**
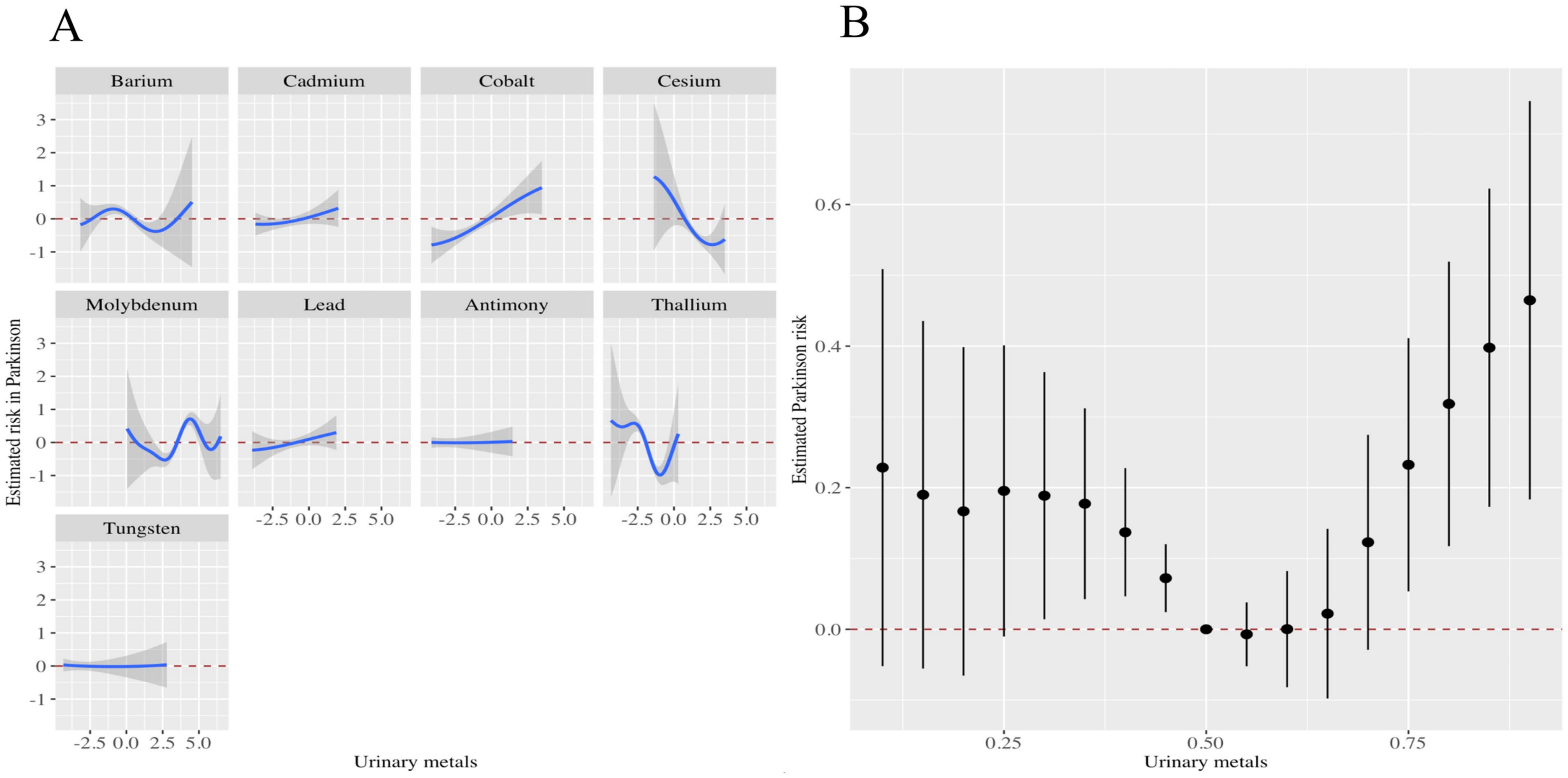
Associations of the urinary metals with PD risk estimated by Bayesian Kernel Machine Regression (BKMR). **(A)** Exposure-response functions were generated for individual metals while holding the concentrations of all other metals constant at their median levels. **(B)** The combined effects of urinary metal mixtures on PD risk were illustrated. This plot presented the estimated difference in PD risk along with the 95% confidence interval when all metal concentrations were set at specific percentiles relative to their medians. The models were adjusted for potential confounders, including gender, age, race/ethnicity, education level, PIR, marital status, BMI, MET, alcohol consumption, smoking status, and CCI.

In addition, the metals with the highest weights in the WQS model were Mo (56.79%), Co (34.20%), Ba (3.33%), and Tu (3.27%) (Figure 2). Next, we showed the relationship between mixed metals and PD risk by BKMR model analysis (Figure 3). Interestingly, we found that most single metals were positively associated with PD, but the relationship with PD risk fluctuated with the change of metal Mo concentration (Figure 3A), and this trend was similarly reflected in mixed metals and PD risk (Figure 3B). In conclusion, although there were fluctuations in the trend of some metals and PD risk, there was a positive association between urinary metals and PD in general.

### Nonlinearity analysis using RCS

**Figure 4:**
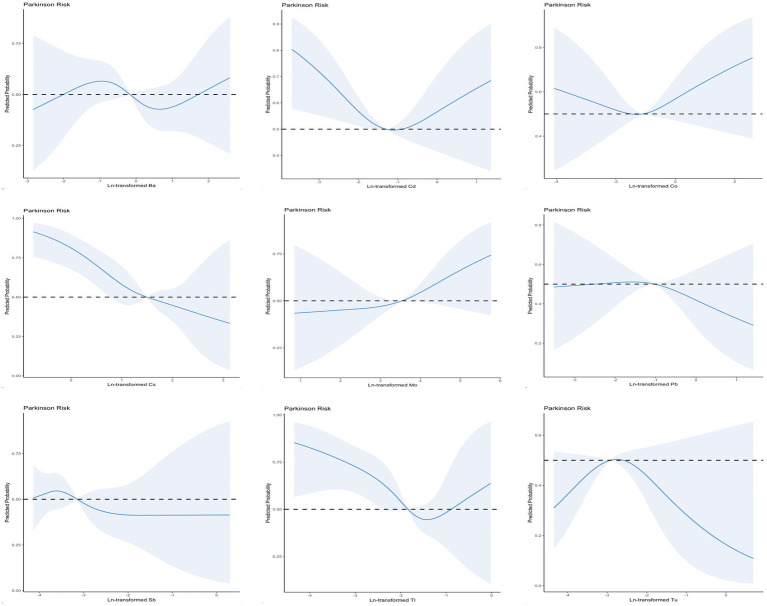
The non-linear relationship between Ln-transformed urinary metals and PD.

To explore the association between urinary metals and the incidence of PD, we analyzed the nonlinear relationship between metal concentrations and the probability of PD using smoothing curve fitting with the penalty spline method (Figure 4). After adjusting for potential confounding factors, including age, sex, race, education level, marital status, family income, body mass index, smoking status, alcohol consumption, and comorbidity index, restricted spline analysis revealed that the probability of PD increased with rising concentrations of Ba, Cd, Mo, Tl, and Co. The rate of change was initially slow but accelerated as the concentrations increased. With the increase of Sb and Pb concentrations, the probability of PD increased, and then gradually tended to be stable. With the increase of Cs and Tu concentrations, the probability of PD gradually increased and then decreased.

### Mediation analysis of CCI

In addition, based on the above research, we analyzed the role of the CCI as a potential mediator in the relationship between urinary metal exposure (Tungsten and Cadmium) and PD (Supplementary Table S3). Through mediation analysis, we found: Tungsten: The indirect effect (ACME) estimates were −0.000000216 (control) and −0.000005519 (treated), with *p*-values of 1, indicating no significant mediation effect through the CCI. In contrast, the direct effect (ADE) estimates were 0.001231822 (control) and 0.001231518 (treated), both significant (*p* < 0.001). These results suggest that Tungsten’s impact on PD is driven primarily by a direct effect rather than through the CCI as a mediator. Cadmium: The indirect effect (ACME) estimates were 0.0000859 (control) and 0.0000939 (treated), with *p*-values of 0.42, indicating no significant mediation through the CCI. Furthermore, the proportion mediated (the proportion of the total effect explained by the mediator) was 0.0479975 (control) and 0.0524939 (treated), with p-values of 0.71, which were not significant. Consequently, Cadmium’s influence on PD was neither direct nor mediated through the CCI (Supplementary Table S3).

Based on these findings, the CCI does not appear to play a significant mediating role in the relationship between Tungsten or Cadmium and PD. The effects of these metals on PD are likely mediated through other pathways.

## Discussion

The current study aimed to evaluate the association between urinary metal concentrations and PD risk among US adults by utilizing the NHANES dataset from 2013 to 2020. In the fully adjusted model accounting for potential confounders, we observed that higher concentrations of urinary Mo, Cd, and Tu were significantly associated with higher odds of PD. The mixed metal analysis consistently showed a positive association between aggregate metal concentrations and PD risk. Further WQS regression and BKMR models suggested metal mixtures containing Mo, Co, Ba and Tu imposed higher PD risk. The restricted cubic spline plots generally revealed nonlinear monotonic exposure-response relationships between individual metal concentrations and PD risk.

Several mechanisms may explain the observed links between metals and PD pathogenesis. For instance, metal dyshomeostasis and accumulation in the substantia nigra could elicit oxidative stress, mitochondrial dysfunction, α-synuclein aggregation, microglial activation, dopamine depletion, and eventual selective loss of dopaminergic neurons, which constitute the pathological hallmarks of PD (18, 19). Additionally, metals may disrupt the ubiquitin-proteasome system to hinder degradation of abnormal proteins implicated in PD (20). The associations of individual metals with PD risk are further discussed below.

### Molybdenum

Our study showed that higher urinary Mo was strongly associated with elevated PD risk, which was consistent with previous occupational investigations. A cohort study of Finnish men revealed increased PD mortality in Mo miners and processing plant workers who suffered high levels of exposure (21). Possible mechanisms include Mo-induced mitochondrial dysfunction, oxidative damage, α-synuclein aggregation and Lewy body formation (22). However, evidence remains scarce regarding Mo exposure from general populations and PD risk. Given the high weight assigned to Mo in the current mixture analysis, more research is warranted to confirm its role.

### Cadmium

In alignment with our findings, earlier studies utilizing NHANES data have linked higher urinary Cd to increased PD prevalence (23). Cadmium is a widespread environmental pollutant that accumulates in the body over time. Beyond direct neurotoxicity, chronic cadmium exposure may raise PD risk by disrupting the blood–brain barrier and intestinal microbiome-gut-brain axis (24). Cadmium also impedes mitochondrial complexes to trigger oxidative stress and interferes with antioxidant defenses (25). Furthermore, cadmium may drive epigenetic modifications through altering DNA methylation and histone acetylation, thus reprogramming gene expression associated with PD pathogenesis (26).

### Tungsten

We observed elevated PD risk with higher urinary Tu concentration, though previous human evidence remains limited. Earlier animal experiments have shown Tu exposure caused dose-dependent loss of tyrosine hydroxylase positive neurons in substantia nigra, possibly by stimulating microglia, elevating inflammatory factors and free radicals (27). Further research is warranted to characterize Tu exposure and investigate its neurological effects.

### Biological implications of nonlinear trends

Through restricted cubic spline analysis, we observed that metals such as barium (Ba) and cadmium (Cd) demonstrated a slow increase in PD risk at lower concentrations, followed by a sharp rise once a certain threshold was exceeded. This nonlinear pattern likely reflects the dose-dependent toxicodynamics of metals. At lower concentrations, metals may induce mild oxidative stress that triggers cellular protective mechanisms. However, at higher concentrations, oxidative damage surpasses the cellular defense capacity, leading to mitochondrial dysfunction and neuronal damage (18, 19).

Interestingly, elements like cesium (Cs) and tungsten (Tu) displayed an initial increase in PD risk followed by a decline at higher concentrations. This may be due to biphasic effects, where low-level exposure activates antioxidant pathways, whereas high-level exposure overwhelms these systems, resulting in neurotoxicity (23). Such nonlinear trends highlight the need to consider exposure thresholds and dose–response relationships when evaluating the neurotoxic effects of metals.

### Synergistic effects of metal mixtures

Our weighted quantile sum (WQS) regression and Bayesian kernel machine regression (BKMR) analyses revealed that metal mixtures, particularly those containing molybdenum (Mo), cobalt (Co), barium (Ba), and tungsten (Tu), have a stronger association with PD risk compared to individual metals. This indicates that these metals may act synergistically to exacerbate neurotoxic outcomes. For instance: (1) Oxidative Stress and Mitochondrial Dysfunction: Mo and Co may disrupt mitochondrial electron transport chain activity, increasing reactive oxygen species (ROS) production. Concurrently, Cd and Tu may impair mitochondrial membrane potential and deplete antioxidant defenses, amplifying oxidative stress and neuronal damage (22, 25). (2) Protein Misfolding and α-Synuclein Aggregation: Metal mixtures may interfere with protein degradation pathways, such as the ubiquitin-proteasome system, leading to the accumulation and aggregation of α-synuclein, a hallmark of PD pathology (20). (3) Neuroinflammation: Mixed metal exposure may activate microglial cells and stimulate the release of pro-inflammatory cytokines such as IL-1β and TNF-α, contributing to chronic neuroinflammation, which is a key driver of PD progression (27).

### Other metals

Though urinary Co showed a strong weight in the WQS mixture analysis, we did not detect clear links with PD risk in single exposure models, consistent with previous equivocal findings (28). Human studies indicate essential metals like Co often have a small therapeutic window—both deficiency and high exposures may incur adverse effects (29). For toxic metals like Pb and Sb, most occupational investigations associated higher exposures with increased PD risk (30, 31), whereas evidence from general exposure remains inconclusive (32), in line with our observations. Such discrepancies may arise because environmental exposures typically occur at much lower levels. Given most participants had very low exposures near detection limits, power might be insufficient to detect subtle effects. Still, tendency towards higher PD probability at high Pb and Sb levels warrants attention. As for Ba and Cs, associations with PD pathogenesis have been rarely reported previously. The observed nonlinearity and uncertainty warrants validation in future studies.

Mechanistically, considered together, our results highlight the potential neurotoxicity and involvement of environmental metals mixtures in PD etiology. Mixture analysis by WQS regression and BKMR models consistently pointed to joint detrimental effects. Simultaneous exposures to multiple metals like Mo, Co, Ba and Tu may additively or synergistically interact to amplify toxicological effects (33). The complex correlations and interactions between co-occurring metals may lead to non-additive influences not captured in single exposure assessments (34). Hence, evaluating metal mixtures represents a more realistic approach towards quantifying health risks for the general population with blended exposures from diverse sources. Moreover, non-linear monotonic trends suggest potential neurotoxic effects may emerge even below current reference levels. Hence, precautionary measures to minimize exposures are imperative considering metal accumulation and synergies over long-term low-level exposures.

Several strengths enhance the validity of the current study. The NHANES dataset constitutes a large nationally representative sample with comprehensive information on potential confounders allowing adjusted analyses. Moreover, urinary biomarkers indicate internal dose and integrate exposures from multiple sources and routes. State-of-the-art laboratory protocols ensured reliable measurements. Sophisticated statistical techniques like WQS and BKMR enabled realistic mixture assessments. Notably, this constituted the first population-based study to systematically evaluate various metal mixtures in relation to PD risk.

Nonetheless, some limitations should be acknowledged. Cross-sectional design hampers causal analysis, and reverse causation cannot be excluded if early disease processes influence exposure or absorption. Still, considering long preclinical phases, influence may be limited. Misclassification bias might occur as questionnaires provided PD case identification. However, previous validation studies reported high reliability using medication inventories and ICD codes (35). Given low PD prevalence, power constraints might hinder detecting subtle effects. Owing to limited sample sizes among racial subgroups, stratified race-specific analyses were not possible though associations might differ. Additionally, while our study identified significant associations between certain metals (e.g., molybdenum, cadmium, and tungsten) and PD risk, it is important to note that some findings, particularly for Pb and Sb, exhibit considerable uncertainty due to wide confidence intervals. For instance, although Pb and Sb showed a trend toward increased PD risk in the higher exposure quantiles, the confidence intervals were wide and included null values. It is important to emphasize explicitly that this study is cross-sectional in nature, based upon data derived from NHANES. Although significant associations between urinary metal concentrations and PD risk were observed, the cross-sectional design inherently precludes definitive causal inference. Therefore, the observed associations indicate correlation rather than causation, and we cannot ascertain the temporal sequence between metal exposure and PD onset. Reverse causation remains possible, as early pathological or physiological processes of PD may influence metal metabolism or excretion, potentially altering urinary metal concentrations. Future research, particularly prospective cohort studies or longitudinal analyses, will be crucial to clearly establish temporal relationships and causality between environmental metal exposure and PD risk. Furthermore, incorporating genetic susceptibility factors and lifestyle variables into longitudinal studies would offer a more comprehensive understanding of the mechanisms linking metal exposure to PD development. By considering the interplay between genetic predisposition, environmental exposures, and lifestyle factors, future research could better elucidate the complex pathways contributing to PD risk and progression.

In summary, our study suggested elevated PD risk with higher urinary concentrations of metals like Mo, Cd and Tu among US adults, which appeared most pronounced at high exposure levels. Mixed exposures to metal combinations seemed to jointly increase PD probability. The associations may be attributable to metal-induced oxidative stress, mitochondrial dysfunction, protein aggregation and neuroinflammation. Considering metal accumulation and synergistic actions, stringent policies to control environmental releases and human exposures are imperative to mitigate potential health risks. Nonetheless, longitudinal cohort studies with incident cases are warranted to establish temporality and elucidate exposure-response relationships over the life course integrating genetic and lifestyle effect modifications. Future research could determine threshold limits and characterize high-risk subgroups to strategize prevention.

## Conclusion

In conclusion, this cross-sectional analysis of NHANES data indicates that higher urinary concentrations of metals including Mo, Cd, and Tu are associated with increased odds of PD among US adults. Mixed exposures to several metals may jointly elevate PD risk in a dose-dependent manner. These links could be mediated through metal-induced oxidative stress, mitochondrial dysfunction, protein misfolding and inflammation. Considering bioaccumulation and interactions, stringent policies should control environmental releases and human exposures to mitigate potential health consequences. Nonetheless, the cross-sectional nature limits causal analysis. Prospective investigations are warranted to characterize exposure-response trajectories and identify intervention opportunities to strategize prevention.

## Data Availability

The original contributions presented in the study are included in the article/Supplementary material, further inquiries can be directed to the corresponding author.
