# Supplementary material for: Urinary metal levels and their association with Parkinson’s disease risk: insights from NHANES 2013–2020
Source: Front Public Health. 2025 Mar 26;13:1439325. doi: 10.3389/fpubh.2025.1439325 (PMC11979115; doi:10.3389/fpubh.2025.1439325)
Supplement: Supplementary file 1 [file Data_Sheet_1.docx]

Supplementary Material

Table S1. Distributions of metals in the study population.

Table S2. Multicollinearity Assessment.

Table S3. Mediation Analysis of the Charlson Comorbidity Index (CCI) in the Association Between Urinary Metal Exposure and Parkinson's Disease.

Figure S1. Pearson’s correlation matrix among Ln-transformed urinary metals in the study population.

Table S1. Distributions of metals in the study population.

| Metals (μg/L) | Detection rate (%) | Median | Interquartile range |
| --- | --- | --- | --- |
| Ba | 100.00 | 0.85 | 0.42-1.86 |
| Cd | 100.00 | 0.27 | 0.14-0.54 |
| Co | 100.00 | 0.33 | 0.18-0.55 |
| Cs | 100.00 | 4.45 | 2.73-6.70 |
| Mo | 100.00 | 34.41 | 17.70-59.70 |
| Pb | 100.00 | 0.36 | 0.20-0.64 |
| Sb | 100.00 | 0.04 | 0.02-0.07 |
| Tl | 100.00 | 0.16 | 0.09-0.24 |
| Tu | 100.00 | 0.05 | 0.03-0.10 |

Table S2. Multicollinearity Assessment.

| **Variable** | **GVIF** | **Df** | **GVIF^1/(2*Df)** |
| --- | --- | --- | --- |
| Barium | 1.455969 | 1 | 1.206635 |
| Cadmium | 2.237529 | 1 | 1.495837 |
| Cobalt | 1.670344 | 1 | 1.292418 |
| Cesium | 3.965024 | 1 | 1.991237 |
| Molybdenum | 2.633267 | 1 | 1.622735 |
| Lead | 2.597719 | 1 | 1.611744 |
| Antimony | 1.994134 | 1 | 1.412138 |
| Thallium | 3.114451 | 1 | 1.764781 |
| Tungsten | 2.180936 | 1 | 1.476799 |
| Age | 1.473227 | 1 | 1.213766 |
| Gender | 1.264422 | 1 | 1.124465 |
| BMI | 1.320989 | 1 | 1.149343 |
| Smoke | 1.836233 | 2 | 1.164078 |
| Alcohol | 1.376329 | 3 | 1.054679 |
| Race | 1.422734 | 4 | 1.045058 |
| Poverty | 1.232859 | 1 | 1.110342 |

GVIF, Generalized Variance Inflation Factor. Df, Degrees of Freedom. GVIF^1/(2*Df), the standardized form of GVIF, allowing the multicollinearity influence of multilevel categorical variables to be compared directly with that of continuous variables.

Table S3. Mediation Analysis of the Charlson Comorbidity Index (CCI) in the Association Between Urinary Metal Exposure and Parkinson's Disease.

| Exposure | Effect Type | Estimate | 95% CI (Lower) | 95% CI (Upper) | p-value |
| --- | --- | --- | --- | --- | --- |
| Tungsten | ACME (control) | -0.000000216 | -0.000026863 | 0.00 | 1 |
| Tungsten | ACME (treated) | -0.000005519 | -0.000051324 | 0.00 | 1 |
| Tungsten | ADE (control) | 0.001231822 | 0.000498571 | 0.00 | <0.001 *** |
| Tungsten | ADE (treated) | 0.001231518 | 0.000497481 | 0.00 | <0.001 *** |
| Tungsten | Total Effect | 0.001231303 | 0.000497770 | 0.00 | <0.001 *** |
| Tungsten | Prop. Mediated (control) | -0.000175048 | -0.016217414 | 0.01 | 1 |
| Tungsten | Prop. Mediated (treated) | -0.000421681 | -0.034078279 | 0.03 | 1 |
| Cadmium | ACME (control) | 0.0000859 | -0.0001598 | 0.00 | 0.42 |
| Cadmium | ACME (treated) | 0.0000939 | -0.0001690 | 0.00 | 0.42 |
| Cadmium | ADE (control) | 0.0016953 | -0.0023258 | 0.02 | 0.52 |
| Cadmium | ADE (treated) | 0.0017034 | -0.0023090 | 0.02 | 0.52 |
| Cadmium | Total Effect | 0.0017892 | -0.0022952 | 0.02 | 0.50 |
| Cadmium | Prop. Mediated (control) | 0.0479975 | -0.4545188 | 0.50 | 0.71 |
| Cadmium | Prop. Mediated (treated) | 0.0524939 | -0.4401019 | 0.50 | 0.71 |


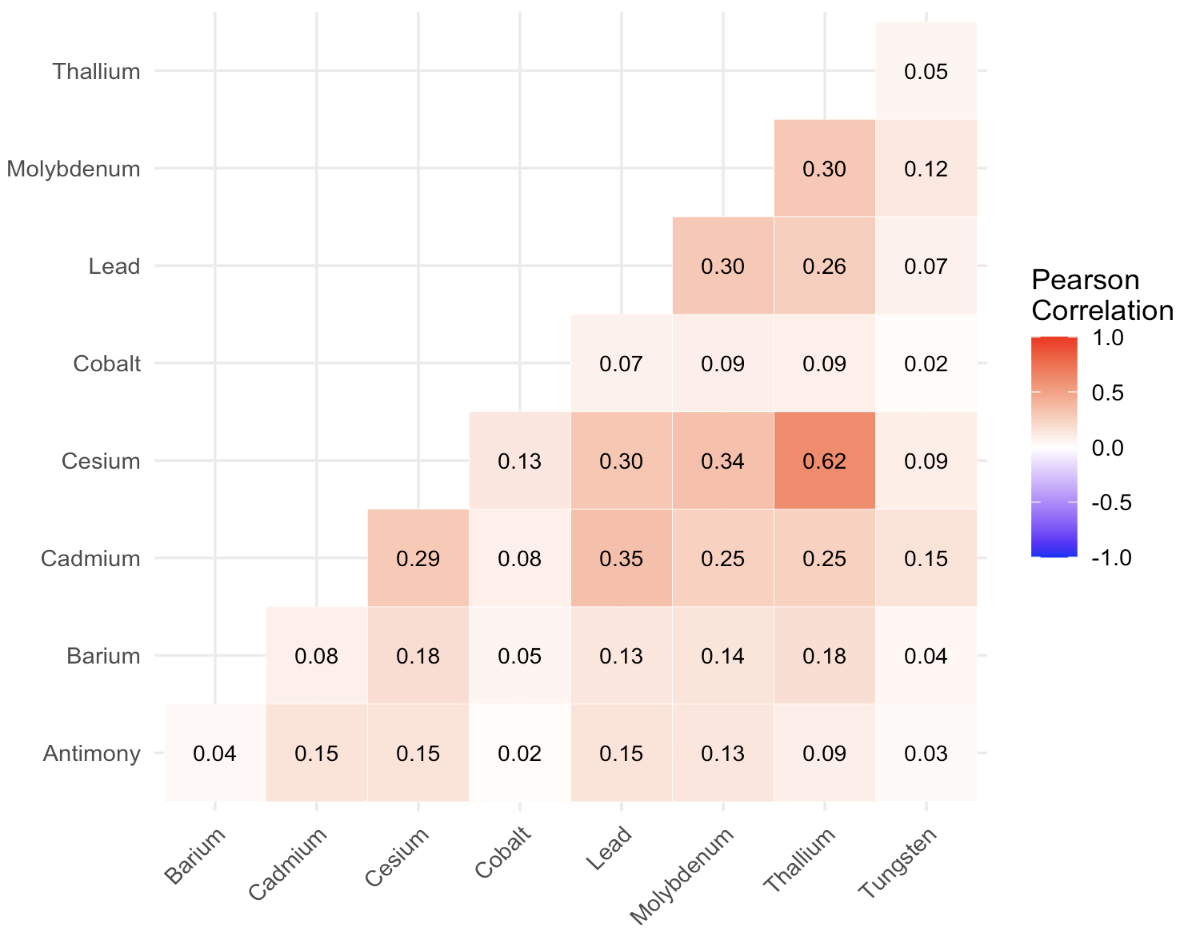


Figure S1. Pearson’s correlation matrix among Ln-transformed urinary metals in the study population.
